# Conformational Ensemble of the Poliovirus 3CD Precursor Observed by MD Simulations and Confirmed by SAXS: A Strategy to Expand the Viral Proteome?

**DOI:** 10.3390/v7112919

**Published:** 2015-11-23

**Authors:** Ibrahim M. Moustafa, David W. Gohara, Akira Uchida, Neela Yennawar, Craig E. Cameron

**Affiliations:** 1 Department of Biochemistry and Molecular Biology, The Pennsylvania State University, University Park, PA 16802, USA; akira.uc@gmail.com; 2 Department of Biochemistry and Molecular Biology, St Louis University School of Medicine, 1100 South Grand Ave, St Louis, MO 63104, USA; sdg0919@gmail.com; 3 Huck Institutes of life sciences, The Pennsylvania State University, University Park, PA 16802, USA; nhy1@psu.edu

**Keywords:** RNA virus, poliovirus, 3CD, polyprotein, MD simulations, conformational dynamics, SAXS

## Abstract

The genomes of RNA viruses are relatively small. To overcome the small-size limitation, RNA viruses assign distinct functions to the processed viral proteins and their precursors. This is exemplified by poliovirus 3CD protein. 3C protein is a protease and RNA-binding protein. 3D protein is an RNA-dependent RNA polymerase (RdRp). 3CD exhibits unique protease and RNA-binding activities relative to 3C and is devoid of RdRp activity. The origin of these differences is unclear, since crystal structure of 3CD revealed “beads-on-a-string” structure with no significant structural differences compared to the fully processed proteins. We performed molecular dynamics (MD) simulations on 3CD to investigate its conformational dynamics. A compact conformation of 3CD was observed that was substantially different from that shown crystallographically. This new conformation explained the unique properties of 3CD relative to the individual proteins. Interestingly, simulations of mutant 3CD showed altered interface. Additionally, accelerated MD simulations uncovered a conformational ensemble of 3CD. When we elucidated the 3CD conformations in solution using small-angle X-ray scattering (SAXS) experiments a range of conformations from extended to compact was revealed, validating the MD simulations. The existence of conformational ensemble of 3CD could be viewed as a way to expand the poliovirus proteome, an observation that may extend to other viruses.

## 1. Introduction

Establishment of a bacterial or viral infection requires that the pathogen be able to evade intrinsic and innate immune defenses of the host cell [1]. Pathogenic bacteria that replicate within cells often encode as many as tens to hundreds “effector” proteins to evade intrinsic and innate immunity [2]. RNA viruses tend to have small genomes, typically encode no more than a dozen proteins in total. This smallness of genome size could be related to the fact that RNA viruses are highly susceptible to mutations [3,4]. RNA viruses developed a strategy to overcome the limited capacity of their encoding genomes and to maximize their functional usability [5,6]. Indeed, these viruses are as efficient as their bacterial counterparts at opposing cellular defense mechanisms.

One well known strategy of RNA viruses is the use of a virus-encoded protease(s) to inactivate host defense proteins, for example pathogen recognition receptors, their adaptors and/or signaling molecules that lead to expression of interferon [7]. These same proteases also enable RNA viruses to express the full complement of its proteome as a single polyprotein that is cleaved co- and/or post-translationally to yield a set of terminally processed proteins [8,9]. These terminally processed proteins often exhibit multiple functions, all of which are generally required for some aspect of virus multiplication [10]. Invariably, cleavage occurs in a manner that yields intermediates: dimeric, trimeric or longer fusion proteins (also referred to here as precursor proteins). Often these precursor proteins actually encode unique functions relative to the fully processed components, thus the use of a polyprotein can actually serve as a strategy and mechanism to expand the proteome [5,6].

**Figure 1:**
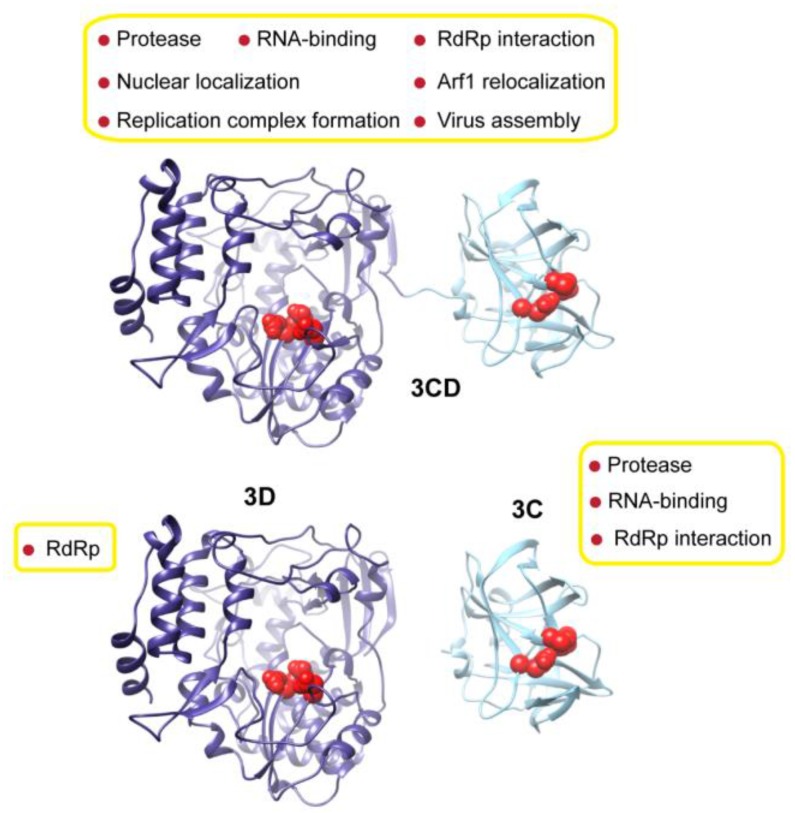
Crystal structure of 3CD is a composite of 3C and 3D proteins. Shown are the crystal structures of the precursor protein 3CD (PDB 2IJD) and the processed proteins 3D (1RA6, blue), and 3C (1L1N, cyan) proteins from poliovirus. The active-site residues of the protease (His-40, Glu-71, Cys-147) and polymerase (Asp-416, Asp-511, Asp-512, 3CD numbering) are shown as red spheres to indicate the relative orientation of the two domains. The different functions associated with the precursor and cleaved proteins are shown next to the structures.

Poliovirus (PV) and other picornaviruses, single-stranded RNA viruses with genome size 7–9 kb [11], use a polyprotein strategy to produce their proteins. The polyprotein is divided into three regions: P1, P2 and P3. The P3 region contains four proteins: 3A, 3B, 3C and 3D. In addition to the fully processed proteins, precursor proteins exist, and some of these accumulate in infected cells. One fusion protein that accumulates is 3CD, a multifunctional protein. PV 3CD consists of the amino acid sequences of both the viral protease 3C (aa 1–183) and the RNA-dependent RNA polymerase (RdRp) or 3D (aa 184–644) [12]. The 3CD protein exhibits protease and RNA-binding activities of 3C, although the specificity may differ [13,14], but the 3CD protein does not exhibit any of the RdRp activity of 3D [15]. In addition, 3CD exhibits numerous activities that are not present in 3C, 3D or the combination of the two individually (Figure 1) [10].

Both 3C and its 3CD precursor perform the majority of proteolytic cleavages of the 247 kDa polyprotein encoded by the poliovirus genome, playing a critical role in regulating the viral gene expression. Notably, 3CD was found to be more efficient (~100–1000 times) and specific than 3C in processing the P1-region of the polyprotein to yield the capsid proteins [13]. For RNA-binding activity, the 3C on its own and in the context of 3CD has been shown to bind the cis-acting replication elements (*cre*) of the viral RNA, forming important interactions required for genome replication; however, 3CD exhibits enhanced ability to form these interactions compared to 3C [16,17,18,19,20,21,22]. As shown in previous studies, 3CD is more efficient than 3C in recognizing the cloverleaf RNA at the 5′-end of the RNA genome, in association with host proteins, to form a ribonucleoprotein complex that is critical for virus replication [17,19,20]. Interactions between 3CD, but not 3C, and the *cre* at the 3′-end of the RNA genome have also been shown [20]. Additionally, 3CD binds to *oriI*, the internal *cre*, with an enhanced specificity compared to that of 3C in the VPg uridylylation reactions [21,22]— incorporation of UMP into VPg protein to form VPg-pUpU that serves as the protein primer for RNA synthesis.

Usually, polyproteins are considered “beads-on-a-string”. For each protein (bead) to be cleaved, the viral protease would require an accessible cleavage site (string). However, with such arrangement, it is difficult to understand how unique function is created. PV 3CD represents a typical example. In the determined 3CD structure [23] (Figure 1), the 3C and 3D domains are arranged as beads-on-a-string, separated by a short linker (aa 180–186) to form an extended conformation with no interactions between the two domains. The precursor is a composite of the structures of 3C [24] and 3D proteins [25,26] (Figure 1). Of note, the linker region between the two domains in the crystal structure appeared weakly ordered, suggesting some flexibility in the 3CD structure [23]. Clearly, the 3CD crystal structure cannot explain how 3C-3D fusion in the precursor protein resulted in enhancing and modulating the protease activity of 3C and abrogating the polymerase function of 3D.

When the structure of PV 3CD was solved, showing a beads-on-a-string organization, the case has been made that dynamics is the missing link between structure and function of viral proteins [27]. Here we use molecular dynamics simulations of PV 3D to determine the extent to which precursor dynamics contributes to the unique activities of this protein relative to its processed components. These studies reveal a compact form of 3CD. The two domains collapse, forming an interface and masking the cleavage site. Interestingly, multiple orientations of the 3C–3D interface in 3CD were observed. The compact conformation of 3CD perturbed dynamics of the 3D domain, for example opening and closing of the nucleotide channel, thus explaining the absence of RdRp activity in the precursor. A single amino acid change in the interface produced a conformational ensemble distinct from the wild-type protein. Small angle X-ray scattering was used to demonstrate the existence of compact conformations in solution. We propose that the conformational dynamics of a viral precursor protein, coupled with the production of new ensembles by single amino acid substitutions may represent a strategy for viruses to expand their proteome/interactome. Studying interdomain interactions in these validated target precursor proteins in RNA viruses generates opportunities to develop new drugs against viral infections.

## 2. Materials and Methods

### 2.1. Molecular Dynamics Simulations

MD simulations were performed using Amber12 software suite [28] and parameters from amber99SB force field as described previously [29]. The starting coordinates for MD simulations were prepared from the crystal structure of 3CD (PDB 2IJD) [23]. The mutated residues in the crystal structure that were engineered into 3CD to facilitate crystallization were mutated back to wild-type residues. The crystal structure has two monomers 1 and 2 in the asymmetric unit that differ in the region encompassing residues 181–184 of the linker; monomer 1 is relatively more extended than monomer 2 by ~1 Å. Independent simulations for the two monomers were carried out. We also carried out a simulation on the 3CD crystal structure without modifying its sequence to wild-type protein. For all simulations, the prepared 3CD monomers were immersed in a truncated octahedral cell filled with TIP3P water molecules. The solvent molecules in the unit cell extended to at least 12 Å from any protein atoms; counterions were added to neutralize the system charge. Simulations were conducted under NPT conditions of constant temperature (300 K) and pressure (1 atm); Berendsen thermostat was used to maintain the constant temperature. Simulations were conducted for a total of 100 ns (wild-type 3CD, monomer 1), 100 ns (WT 3CD, monomer 2), and 50 ns (mutant 3CD structure), using 1 fs integration time-step; SHAKE algorithm was employed to constrain bonds involving any hydrogen atoms. Periodic boundary conditions were employed to calculate non-bonded interactions using 9 Å cutoff distance. The Particle Mesh Ewald (PME) method was used to calculate electrostatic interactions. Snapshots of the simulated structure were taken at 1 ps interval. Analysis of the MD trajectories was done using PTRAJ and CPPTRAJ of the Amber suite [30].

To enhance the conformational sampling of 3CD, we carried out accelerated MD (aMD) simulations of WT 3CD (monomer 1) using the GPU version of pmemd in Amber 14 [31], modified to support the rotatable accelerated molecular dynamics-dual boost (RaMD-db) procedure described by Doshi and Hamelberg [32]. The structure was solvated in TIP3P water with 15 Å buffer, minimized and subjected to 10 ns of conventional MD using Amber ff14SB force field under NPT conditions with a 2 fs time-step. Boosting parameters for the RaMD-db run were calculated from the equilibration run according to the procedures described for traditional aMD in the Amber manual. The RaMD-db calculation was switched to constant temperature and volume (NVT) and run for an additional 170 ns of simulation time with snapshots saved every 10 ps. The resulting trajectory was analyzed using CPPTRAJ.

For free energy calculations of the interdomain interactions, we used SITRAJ program [33,34]. The program calculates ΔG for selected snapshots as the sum of (i) van der Waals interactions; (ii) Coulomb interactions; (iii) change in reaction field energy that is determined by solving Poisson-Boltzmann equation; and (iv) non-polar solvation energy that is proportional to the solvent accessible solvent area. The calculated ΔG is scaled by an empirical parameter as a crude treatment for entropy-enthalpy compensation [34,35]. The calculations were carried out on 5000 snapshots across the last 50 ns of the MD trajectory of the wild-type simulation (monomer 1). The selected snapshots were first clustered into six groups by PTRAJ using means algorithm [36] to make sure that ΔG is calculated over parts of the trajectory that have small fluctuations of the root-mean-square deviations (RMSD); which is recommended by SITRAJ developers. The estimated ΔGs for the different clusters were obtained by averaging of 530, 611, 1008, 645, 874, and 1332 calculations (corresponding to the number of snapshots in the clusters 1 to 6, in the same order). Knowing the limitations and caveats in this type of free energy calculations, we calculated ΔG for structures corresponding to the beads-on-a-string conformation of 3CD, where there is no interdomain interactions, and used it as a reference to calculate ΔΔG for the different clusters. The reported ΔΔG represents the favorable interdomain interaction in compact 3CD conformations sampled during simulations relative to the beads-on-a-string extended conformation shown by X-ray crystallography.

### 2.2. SAXS Experiments

#### 2.2.1. Expression and Purification of 3CD

3CD protein was expressed using SUMO-fusion system. The poliovirus 3CD gene, containing the same series of mutations in the 3CD construct utilized for crystallization [23], was subcloned into the expression plasmid pET24-6His-SUMO and transformed into Rosetta(DE3) competent cells [37]. The cells containing the fusion plasmid were grown at 30 °C overnight in 100 mL of NZCYM medium supplemented with kanamycin at 25 μg/mL (K25), chloramphenicol at 20 μg/mL (C20), and dextrose at 0.4%. The overnight cell culture was used to inoculate 2 liters of NZCYM autoinducing medium supplemented with K25 and C20; cells were grown at 37 °C to an OD_600_ of ∼1.0 and then growth continued at 25 °C for an additional 16 h before harvesting the cells. Cell pellets were washed once with buffer containing 10 mM Tris, pH 8.0, and 1 mM EDTA, suspended in lysis buffer [100 mM potassium phosphate, pH 8.0, 500 mM NaCl, 5 mM imidazole, 1.0 mM EDTA, 20% glycerol, 10 mM β-mercaptoethanol (β-ME), 2.8 μg/mL pepstatin A, and 2.0 μg/mL leupeptin], and disrupted by passage through a French pressure cell at 20,000 psi. Phenylmethanesulfonyl fluoride (PMSF) and Nonidet P-40 were added immediately to the cell lysate to final concentrations of 1.0 mM and 0.1%, respectively. Polyethyleneimine was added slowly to the cell lysate, to precipitate nucleic acid, at a concentration of 0.025%. The lysate was stirred at 4 °C for 30 min and then centrifuged at 25,000 rpm; the clear solution was retained, to which ammonium sulfate was added slowly to reach 60% saturation. The ammonium sulfate suspension was pelleted by centrifugation at 25,000 rpm for 30 min; the pellet, containing 3CD protein, was re-suspended in buffer A (50 mM Tris, pH 8.0, 500 mM NaCl, 10% glycerol, and 10 mM β-ME) containing 5 mM imidazole. The protein sample was loaded onto an Ni-nitrilotriacetic acid column (Ni-NTA, ∼1 mL bed volume per 25 mg total protein) that was pre-equilibrated with buffer A containing 5 mM imidazole. The column was washed to baseline with the equilibrating buffer and eluted with a linear imidazole gradient (50 to 500 mM) in buffer A. Fractions containing 3CD protein, checked by SDS-PAGE, were pooled, Ulp1 protease was added to cleave the SUMO tag (∼1 μg protease per 1.0 mg SUMO-fusion protein), and the sample was dialyzed overnight against buffer A containing 0.5 mM EDTA. The dialyzed sample was further fractionated on a HiLoad 26/60 Superdex 200 gel filteration column, equilibrated with buffer B [50 mM Tris, pH 7.5, 200 mM NaCl, 10% glycerol, 0.5 mM EDTA, and 2 mM dithiothreitol (DTT)]. Fractions containing 3CD protein were pooled together and concentrated using Vivaspin concentrator (MWCO 10,000) to a final concentration of 5 mg/mL.

#### 2.2.2. SAXS Data Collection and Analysis

Samples of 3CD protein at concentrations of 0.54, 1.1, 2.2, and 4.3 mg/mL were prepared in a buffer containing (50 mM Tris, pH 7.5, 200 mM NaCl, 5% glycerol, 2 mM DTT, and 0.5 mM EDTA). Monodispersity of samples was checked by DLS. Synchrotron X-ray scattering data were collected at MacCHESS on the G1-line station. SAXS data were collected at 293 K using dual PILATUS 100K-S detector and a wavelength of 1.224 Å. The setup of the sample-to-detector distance facilitated simultaneous small- and wide-angle data recording, covering a momentum transfer range (*q*-range) of 0.01 < *q* < 0.8 Å^−1^ [*q* = $\frac{4\text{πsin}\left(\text{θ}\right)}{\text{λ}}$, where 2θ is the scattering angle]. Exposure times of 1 min in fifteen 4-seconds frames were used for the measurements; this allowed monitoring for any radiation damage effect. No radiation damage was detected. The RAW software was used for initial data reduction and background subtraction [38]. The data at high concentrations showed some concentration dependence and a combined scattering curve was prepared from data recorded at low and high concentrations. The forward scattering *I*(0) and the radius of gyration (*R_g_*) were calculated using the Guinier approximation, which assumes that at very small angles (*q* <1.3/*R_g_*) the intensity is approximated as *I*(*q*) = *I*(0)$\text{exp}\left[-\frac{{\left(qR_{\text{g}}\right)}^{2}}{3}\right]$. GNOM [39] was used to calculate the pair-distance distribution function P(r), from which the maximum particle dimension $\left({\text{D}}_{\text{max}}\right)$ and ${\text{R}}_{\text{g}}$ were determined. The molecular mass was estimated using (i) comparison with lysozyme standard protein; (ii) Porod invariant [40]; and (iii) SAXS MoW web tool [41]. *Ab initio* low-resolution models were reconstructed using DAMMIN [42] for data in the range (0.012 < *q* < 0.4 Å^−1^) and GASBOR [43] for data in the range (0.012 < *q* < 0.5 Å^−1^). Ten models were generated from each program and averaged using DAMAVER [44]. The normalized spatial discrepancy parameter (NSD) obtained from DAMAVER indicated the similarity between models used for average calculations. NSD values ≤ 1.0 are expected for similar models. The theoretical scattering profiles of the constructed models were calculated and fitted to experimental scattering data using CRYSOL [45] and FoXS [46]. The 3CD structures from X-ray and MD simulations were fitted into the SAXS model using SUPCOMB [47].

To evaluate the conformational flexibility of the two domains in the protein, Ensemble Optimization Method (EOM 2.0) was used [48,49]. This method assumes the existence of a mixture of conformations in solution; the average scattering of the mixture fits the experimental data. In EOM, a random pool of 10,000 conformers of 3CD was generated; in these conformers the linker residues were allowed to have random-coil conformations and 3C-domain assumed different conformations relative to a fixed 3D-domain. The theoretical scattering was calculated for each generated model by CRYSOL. Using genetic algorithm in GAJOE, 100 sub-ensembles with varying numbers of conformers were selected. The distribution of *R*_g_ of the initial pool was compared with the corresponding distributions of the 100 sub-ensembles and the one with the best discrepancy (χ-value) was reported as the best solution. Information about conformers constituting the selected best sub-ensemble and their contributions to scattering was obtained. The EOM calculations were repeated three times; in each the same results were found.

#### 2.2.3. Dynamic Light Scattering

Dynamic light scattering (DLS) experiments of the expressed and purified 3CD protein (2.0 mg/mL) were performed using Viscotec 802 instrument at 293 K. The DLS data were processed by OmniSIZE 3.0 software to get an estimate for the hydrodynamic radius ${\text{R}}_{\text{h}}$ and polydespercity of the protein sample. The estimated ${\text{R}}_{\text{h}}$ was 38 Å, and the estimated polydespersity was ∼22.5%; see Figure S1.

## 3. Results

### 3.1. MD Simulations Revealed Domain Motions and Formation of Interdomain Interactions in 3CD

Conformational dynamics of 3CD was investigated using MD simulations as described in the Experimental section §2.1. MD simulations permit the study of time-dependent structural changes of proteins, providing detailed information on protein conformations that are relevant to function. The structure of the wild-type (WT, monomer 1) 3CD was immersed in a box containing water solvent and counterions were added to neutralize charges on the protein. The whole system was subjected to a 100 ns all-atom MD simulation with structural snapshots stored every 1 ps. Analysis of the trajectory of the MD simulation yielded detailed information on the dynamics of 3CD.

**Figure 2:**
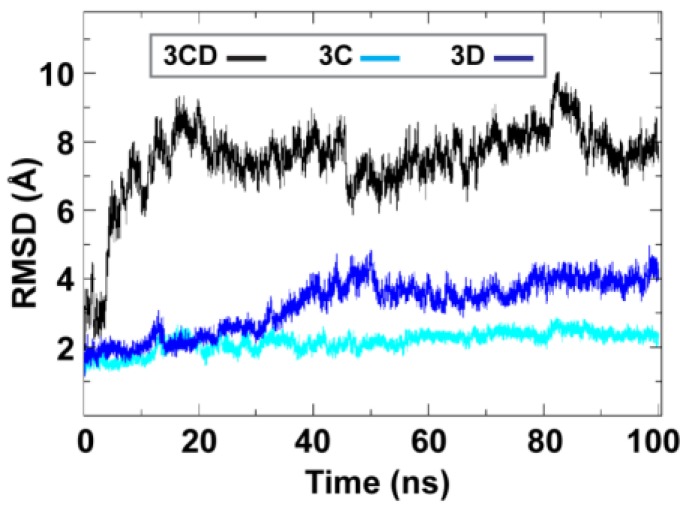
Root-mean-square deviations (RMSD) analysis of the molecular dynamics simulation (MD) trajectory reveals large domain movements relative to crystal structure. The RMSD for the backbone atoms of the full-length 3CD (black), of the domains 3C (cyan) and 3D (blue) are plotted as a function of time. The RMSD calculated for 3C and 3D domains showed much lower values than that calculated for 3CD. The dramatic increase in RMSD values for the full-length 3CD during the first 10 ns of the simulations suggested a large domain motion of the precursor protein. Moreover, the lower RMSD values of 3C-domain compared to that of 3D-domain indicated less dynamics of 3C-domain compared to 3D-domain.

To obtain information on the extent of structural changes of 3CD during simulations, we calculated the root-mean-square deviations (RMSD) of all snapshots in the MD trajectory relative to the starting coordinates. The calculations were carried out utilizing backbone atoms of all residues in the protein and backbone atoms of the individual 3C and 3D domains (Figure 2). Clear differences were observed between the RMSD calculated using the 3CD precursor protein and that calculated for the individual domains. In the case of the precursor protein, the RMSD showed a dramatic increase during the first 10 ns of the simulation, reached an average of ∼8 Å after 20 ns and continued fluctuating around this average for the rest of the MD trajectory. For 3C-domain, the calculated RMSD fluctuated around an average of ∼2 Å throughout the entire trajectory. For 3D-domain, a gradual increase in RMSD values was observed during the first half of the MD trajectory, reaching a value of ∼4 Å and fluctuating around this average value for the remainder of the simulation. This large discrepancy in RMSD values of the individual domains compared to that of the precursor protein suggested large domain movements during the simulation. Also, the larger RMSD values of the 3D-domain compared to that of 3C-domain (∼4 Å *vs.* ∼2 Å) indicated that 3D-domain is more dynamic than 3C-domain.

**Figure 3:**
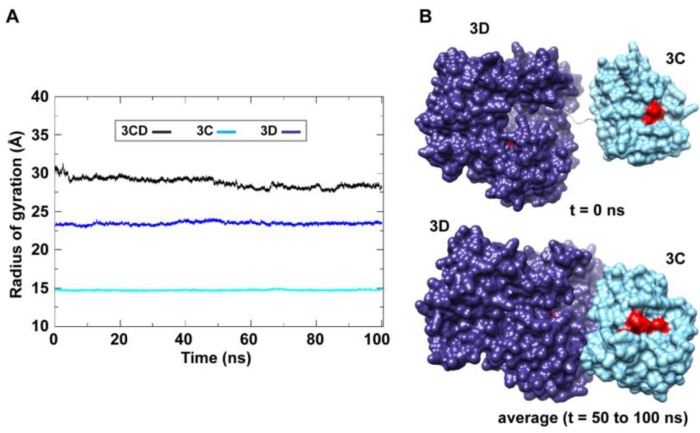
MD simulations reveal interdomain interactions in 3CD. (**A**) The radius of gyration (*R_g_*) of the full-length 3CD protein (black) and the individual domains 3C (cyan) and 3D (blue) are plotted as a function of time. The *R_g_* of 3CD substantially decreased during the simulation, whereas the *R_g_* of 3C-domain remained almost constant and the *R_g_* of 3D-domain showed very small variation. The decrease in *R_g_* values of 3CD reflects the development of the interactions between 3C and 3D domains during simulations. (**B**) Shown are the 3CD structure at the starting time of the simulation (*t* = 0) and the average structure calculated over the last 50 ns of the trajectory of monomer 1. The structures are rendered as surface, the 3C and 3D domains are colored cyan and blue, respectively. The active-site residues of the protease (His-40, Glu-71, Cys-147) and polymerase (Asp-416, Asp-511, Asp-512) are indicated by red color to show the relative orientation of the two domains. The starting 3CD structure adopts an extended conformation with no interdomain interactions in contrast to that observed in the simulated average structure.

The large domain movements in 3CD indicated by RMSD analysis (Figure 2) was readily observable by visual inspection of the MD trajectory using the molecular graphics software CHIMERA [50]. The 3C and 3D domains evidently approached each other early during simulations to form a compact conformation. To assess this observed conformational change in a more quantitative manner, the radius of gyration (*R*_g_), which describes the root-mean-square distance of the protein atoms from their common center of mass, was calculated as a function of time. The *R*_g_ calculations were carried out for the full-length 3CD protein as well as for the individual 3C and 3D domains (Figure 3A); only backbone atoms were used in the calculations. For 3C-domain, the calculated *R*_g_ remained almost constant throughout the simulation with an average value of 14.7 ± 0.06 Å. The calculated *R*_g_ of the 3D-domain showed more variation than that of the 3C-domain, fluctuating around an average value of 23.4 ± 0.2 Å. For 3CD, the calculated *R*_g_ exhibited substantial variation; it decreased by approximately 8% from 30.1 Å at the starting time of simulation to an average of 27.8 ± 0.6 Å over the last 50 ns of the simulation. The decreasing *R*_g_ of the full-length 3CD without significant changes in the *R*_g_ values of the individual 3C and 3D domains is consistent with the large domain movements hinted by RMSD analysis and that the two domains are getting closer during the simulation. Calculating the average structure of 3CD using structural snapshots from the last 50 ns of the MD trajectory clearly revealed a compact conformation with interactions between 3C and 3D domains relative to their extended conformation in the crystal structure (Figure 3B). The 3C-domain moved approaching the back of the 3D-domain, towards the NTP channel of the polymerase.

**Figure 4:**
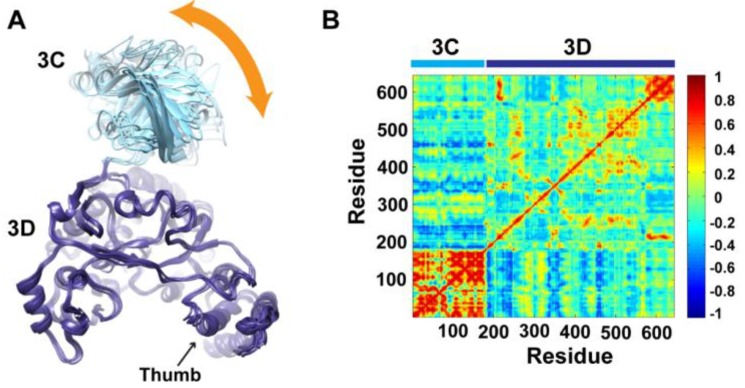
Conformational changes of 3CD during simulations. (**A**) Representative conformations of the clusters (1–6) sampled during the last 50 ns of wild-type (WT) 3CD simulation of monomer 1, superimposed using Cα atoms of 3D-domain. The positioning of 3C-domain (cyan) relative to 3D-domain (blue) varies among the different conformations, resulting in small variations at the interface between the two domains as indicated by the orange arrow. (**B**) Dynamic Cross-Correlation Map (DCCM) analysis of the last 50 ns, calculated for Cα atoms of all residues in 3CD. Residues of 3C-domain exhibited strong correlations, indicating rigid-body motion of the domain. Also, residues of the thumb (aa 563–644) in the 3D-domain showed strong correlations; which is consistent with the slightly larger deviations observed for the thumb residues shown in (**A**).

To determine whether the arrangement of the 3C and 3D domains in the average MD structure corresponds to a single unique conformation or to multiple conformations sampled during simulations, we performed cluster analysis. The structural snapshots of the last 50 ns of the MD trajectory can be grouped into six clusters (1 to 6). Representative conformations of the clusters are shown in Figure 4A. Superpositioning of conformations of the different clusters using backbone atoms of the 3D-domain revealed relatively large conformational changes for residues of the 3C-domain. Also, small conformational changes for residues of the thumb (aa 563–644) in the polymerase domain were noticed. Inspection of these different conformations showed that they are similar to the compact conformation of the average structure (Figure 3B). Apparently, in all conformations, the same faces of the 3C and 3D domains are involved in interdomain interactions. Nevertheless, the details of these interactions vary from one conformation to another, suggesting a dynamic interface. Thus, a characteristic feature of the conformational changes observed in the simulations of 3CD is a large movement of the 3C-domain towards the 3D-domain with variations in the relative positions of the two domains. It is worthy to note that the collapsing of the two domains in 3CD structure to form a compact conformation occurred early during the first 10 ns of the simulation.

To obtain information on the nature of motions of the two domains in 3CD and their correlations, we analyzed the correlations among positional fluctuations of Cα atoms in the simulated structure, or what is known as dynamic cross-correlation map (DCCM). In this analysis, residues that move in the same direction appear positively correlated whereas residues moving in opposite directions appear negatively correlated. Figure 4B shows the calculated DCCM for residues of 3CD using the last 50 ns of the MD trajectory. The residues of the 3C-domain exhibited strong intradomain positive correlations (red color in the calculated map), which can be interpreted as an indication of a collective movement of the whole domain. Also, the thumb residues (aa 563–644) of the polymerase domain showed strong positive correlations with each other, suggesting their collective movement as well. These collective movements of the thumb residues and 3C-domain are in agreement with the conformational changes observed by cluster analysis (Figure 4A).

Of note, the intradomain correlations of 3D-domain differ significantly from the correlations reported previously in the MD simulation study of the cleaved polymerase [29] and protease [51]. The characteristic negative correlations of residues surrounding the template-nascent RNA duplex channel, the template channel and the NTP channel in the 3D^pol^, which indicated the expansion and contraction of these channels of the polymerase, are lacking in the 3D-domain of the precursor protein; additionally, the positive correlations among the functional motifs in the palm and fingers subdomains are less pronounced in the precursor protein compared to the mature polymerase. Inspecting the interdomain correlations revealed that residues of the 3C-domain were negatively correlated (blue color in the map) with regions of the fingers and palm of the 3D-domain encompassing residues 186–190, 238–246, 360–370, and 423–434. These regions of the 3D-domain appeared to be on the same side or near the interface with the 3C-domain. The negative correlations suggest that they move in a direction opposite to that of the 3C-domain movement. Thus, from DCCM analysis it can be concluded that the 3C-domain moves as a whole unit, or what is called “rigid-body” motion, towards a unique face of the 3D-domain. Further analysis of the MD trajectory using principal component analysis (PCA), which filters the noise from major motions in MD simulations as described in our previous study [29], revealed that the rigid-body movement of 3C-domain towards the polymerase domain is the major motion observed during simulations (Figure S2).

**Figure 5:**
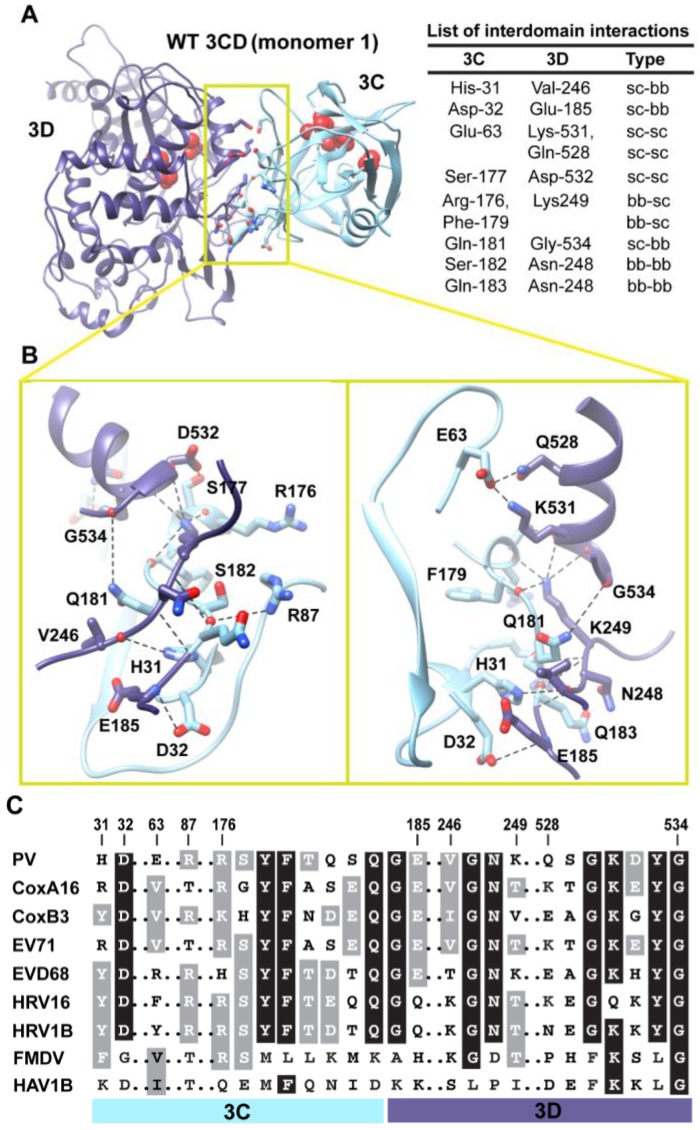
Interactions between 3C and 3D domains in the compact conformation of 3CD (monomer 1). (**A**) Shown is the simulated WT 3CD with interface residues displayed as sticks; the 3C and 3D domains are colored cyan and blue, respectively. The active-site residues of the protease (His-40, Glu-71, Cys-147) and polymerase (Asp-416, Asp-511, Asp-512) are shown as red spheres to help identifying the relative orientation of the two domains. The list of interdomain interactions is shown at the right of the panel; three types of interactions are indicated (sidechain-sidechain, sc-sc; sidechain-backbone, sc-bb/bb-sc; backbone-backbone, bb-bb). (**B**) Two close-up views of the interface marked by the yellow box in (**A**). Interacting residues are shown as sticks and their C-atoms are colored according to the corresponding domains. Interface residues are engaged in many H-bonding interactions and salt bridges, depicted as black dashed-lines. (**C**) Shown is part of the sequence alignment of 3CD proteins from picornaviruses, including poliovirus (PV), coxsackievirus A16 (CoxA16), coxsackievirus B3 (CoxB3), enterovirus 71 (EV71), enterovirus D68 (EVD68), human rhinovirus 16 (HRV16), human rhinovirus 1B (HRV1B), foot-and-mouth disease virus (FMDV), and hepatitis A virus 1B (HAV1B). The residues shown in the alignment are those involved in the interdomain interactions: 31–32, 63, 87, 176–185, 246–249, and 528–534. Many of the interface residues are highly conserved.

### 3.2. Interface Residues and Stability of the Interdomain Interactions

Next, we analyzed the conformations visited during simulations of the WT 3CD to obtain information on residues involved in the interdomain interactions. The 3C and 3D domains bury ∼500 Å^2^ of surface area between them. Residues mediating the interactions between the two domains are shown and listed in Figure 5A,B. The interface is formed by amino acids from the 3C-domain (residues 31, 32 and 63), the linker (residues 176–185), and from the 3D-domain (residues 246–248 of the fingers subdomain and residues 528–534 of motif D in the palm subdomain of the polymerase). The contact surface between the two domains involves electrostatic and weak hydrogen bonding interactions made by backbone and sidechain atoms. The linker region is sandwiched between the two domains to form an intricate network of interactions. The sidechain of Ser-177 forms a hydrogen bond with that of Asp-532. The carbonyl oxygen atoms of four residues, two from each domain, including Arg-176, Phe-179, Asp-532 and Tyr-533, form an oxyanion hole that accommodates the positively charged sidechain of Lys-249. The sidechain of Gln-181 is engaged in hydrogen bonding interaction with the backbone of Gly-534. The backbone atoms of Ser-182 mediate hydrogen bonding interactions with both backbone and sidechain atoms of Asn-248 and Arg-87, respectively. The backbone atoms of Asn-248 in turn are hydrogen bonded to the backbone atoms of Gln-183. The backbone nitrogen atom of Glu-185 forms a hydrogen bond with the sidechain of Asp-32. The residue preceding Asp-32, His-31, mediates a hydrogen bonding interaction between its sidechain and the carbonyl oxygen atom of Val-246. Finally, Glu-63 forms a salt bridge with Lys-531 and a hydrogen bond with the sidechain of Gln-528.

Interestingly, many of the residues at the interface are linked to functions. In the 3C-domain, His-31, Asp-32, Glu-63 and Arg-87 have been shown by NMR to be involved in RNA-binding by 3C [16,51]. Mutations of His-31 and Asp-32 in 3C impair the RNA recognition activity [17]. The residue Arg-176 is also known to be implicated in RNA recognition [24]. In addition, Arg-176 and Phe-179 are involved in peptide binding [51]. In the 3D-domain, Lys-249 (Lys-66 in PV RdRp numbering) is nearby Lys-61 of 3D that was previously shown to be critical for polymerase function [52]. Residues 528–534 correspond to motif D of the polymerase that is also known to be critical for polymerase function [53,54]. Therefore, the interactions between the two domains could be linked to the functional differences between the precursor protein 3CD and its cleaved products.

When we evaluated whether the interdomain interactions in PV 3CD exist in the related 3CD proteins from other picornaviruses (Figure 5C), we found that many of these interactions are conserved but some are not. Interactions in PV 3CD that involve backbone atoms and sidechains of residues at positions 31, 32, 87,177, 181, 249 and 532 are likely to exist in the related proteins; residues at these positions are either conserved or substituted by residues capable of making similar interactions. However, interactions mediated by Glu-63 are predicted to be unique for the poliovirus protein. Our interpretation of this observation is that picornaviral 3CD proteins likely adopt compact conformations in which the two domains interact with each other.

After examining the interface between 3C and 3D domains in the simulated WT 3CD and showing that the contact surface between the two domains involves many interactions, we evaluated the stability of the interdomain interactions in the compact conformation relative to the extended conformation seen in crystal structure. To do so, we carried out the free energy calculations using SIETRAJ as described in the Experimental section 2.1. The relative free energy (ΔΔG) of 3C-3D interactions were calculated for the different conformations visited during simulations; see Table 1. The compact conformations with 3C-3D interdomain interactions were found to be more stable than the extended conformation with non-interacting domains by an average of −7.4 ± 0.89 kcal/mol. Considering the number of interactions between the two domains described above, the estimated stabilization energy seemed reasonable.

**Table 1:** Relative free energy of interdomain interactions in WT 3CD.

Conformation	ΔΔG*^a^* (kcal/mol)
Crystal structure*^b^*	0
Cluster 1	−7.73 ± 0.89
Cluster 2	−8.55 ± 0.98
Cluster 3	−6.81 ± 0.85
Cluster 4	−6.25 ± 0.74
Cluster 5	−8.26 ± 0.90
Cluster 6	−6.84 ± 0.98
average	−7.41 ± 0.89

*^a^* errors correspond to standard deviations; *^b^* used as a reference for the free energy calculations.

**Figure 6:**
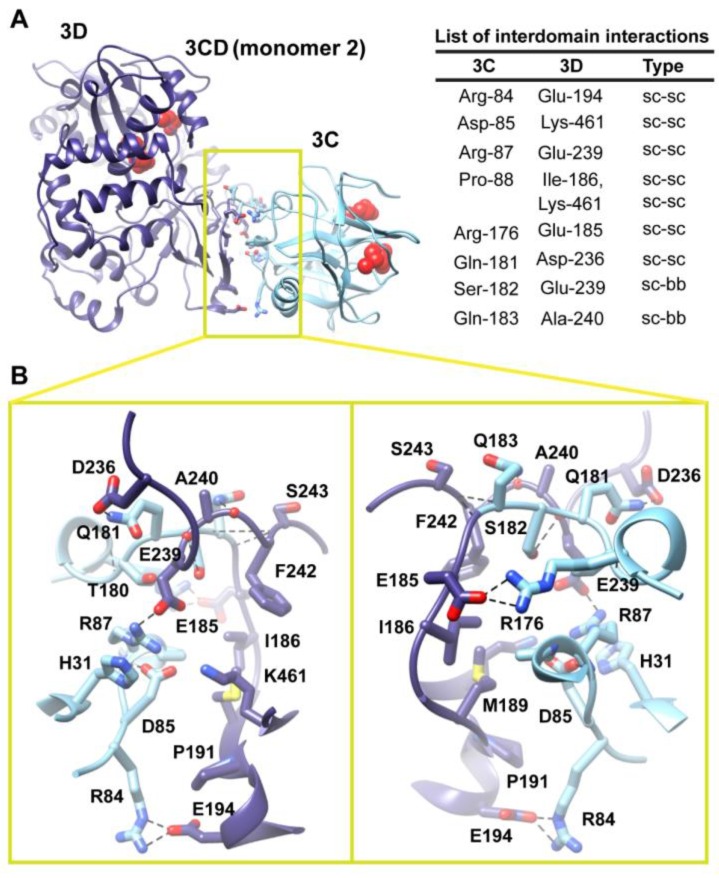
Interface in the compact conformation of 3CD (monomer 2). (**A**) The simulated 3CD is shown with interface residues displayed as sticks; the 3C and 3D domains are colored cyan and blue; respectively. The active-site residues of the protease (His-40, Glu-71, Cys-147) and polymerase (Asp-416, Asp-511, Asp-512) are shown as red spheres to help identifying the relative orientation of the two domains. Interdomain interactions are listed at the right of the panel; two types of interactions are indicated (sidechain-sidechain, sc-sc; sidechain-backbone, sc-bb). (**B**) Two close-up views of the interface marked by the yellow box in (**A**). Interacting residues are shown as sticks and their C-atoms are colored according to the corresponding domains. Interface residues are engaged in many H-bonding (black dashed-lines), hydrophobic and electrostatic interactions. The contact surface between the two domains in the mutant 3CD is distinct from that of monomer 1 shown in Figure 5.

Two monomers were present in the 3CD crystal structure with slightly different conformations of the linker. In order to assess the impact of starting conformation on the outcome of the simulation, we performed a second simulation using monomer 2. Similar to monomer 1 simulation, analysis of the MD trajectory revealed a 3CD molecule forming a compact conformation with a surface area of ∼460 Å^2^ buried between 3C and 3D domains. Interestingly, a new interface in monomer 2 was revealed and appeared to be different from that observed in monomer 1. The 3C-domain moved ~20 Å and rotated ~60° relative to the conformation observed for monomer 1, now reaching the upper part of the fingers in the 3D-domain (Figure S3A,B). The new interface is formed by the loop residues Arg-84, Asp-85, Arg-87 and Pro-88 projected from 3C-domain that interact with residues Ile-186, Glu-194, Glu-239 and Lys-461 from the fingers of 3D-domain (Figure 6). The interface residues from 3D-domain, in turn, interact with residues Arg-176, Gln-181, Ser-182 and Gln-183 at the C-terminus of the 3C-domain. Examination of the interface residues showed that residues at positions 84, 85, 87 and 186 are completely conserved in the related picornaviral proteins; the remaining residues showed a high level of conservation. The site of the 3D-domain contributing to the interface in monomer 2 is adjacent and non-overlapping to that presented in monomer 1. Also, the loop residues 84–88, which are known to play a role in RNA binding [16] in 3C-domain, are adjacent to the 31–32 site that is part of the interface in monomer 1 (Figure 5). The linker residues 176–183 contribute to the contact surfaces in the two monomers. The interfaces in the two monomers are predominately formed by electrostatic and hydrogen bonding interactions. Nevertheless, in monomer 2 there is a hydrophobic contribution to the interface from the interaction between Pro-88 from 3C-domain and Ile-186 from 3D-domain (Figure 6). Thus, it can be argued that 3CD is capable of assuming different compact conformations that could serve distinct functions at different stages of the viral lifecycle.

**Figure 7:**
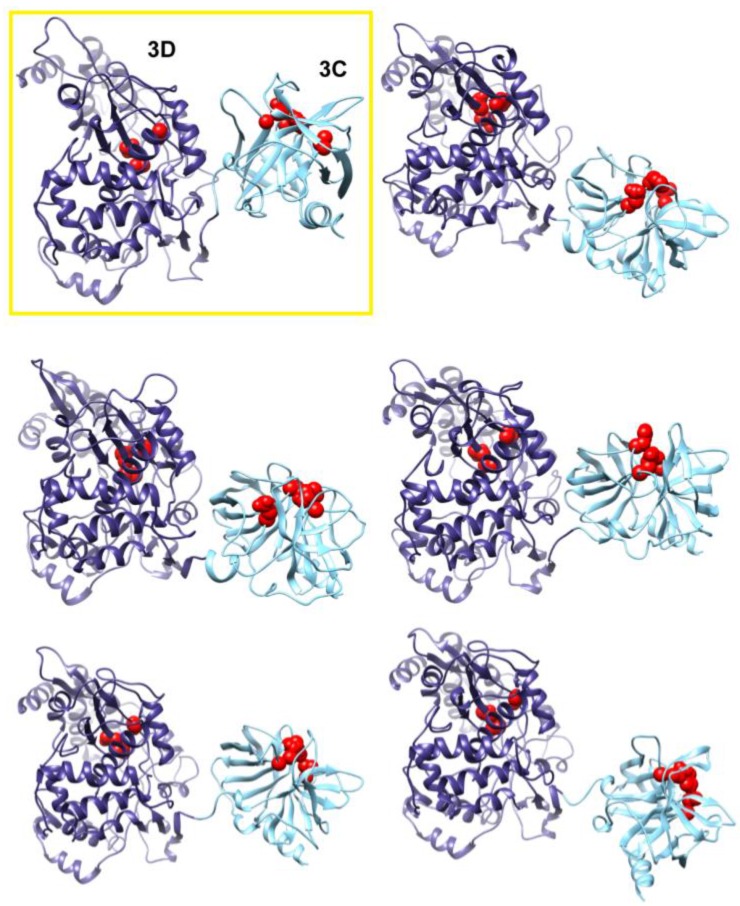
Accelerated MD simulations reveal dynamic interface between 3C and 3D domains. Shown are five representative conformations of 3CD sampled during the accelerated MD simulations (including both compact and extended conformations), revealing the dynamic nature of the interface between the two domains of the protein. The interface in the compact conformation from the conventional MD is shown in a yellow box for comparison. The 3C and 3D domains are colored cyan and blue, respectively; active-site residues of the protease (His-40, Glu-71, Cys-147) and polymerase (Asp-416, Asp-511, Asp-512) are shown as red spheres to help identifying the relative orientations of the two domains.

The observation of two unique orientations of the 3C-domain relative to the 3D-domain in the two simulated 3CD monomers begged the question: do additional conformations exist? To address this question we performed a 170 ns accelerated MD simulation (aMD) as described in the Experimental section §2.1 that enhanced sampling of the conformational space of 3CD. Similar to the above two simulations, the 3CD assumed compact conformations with interfaces that overlap with the interfaces observed in the conventional MD simulations (Figure 7). The switching among different conformations is shown in movie S1. The buried surface area between the two domains was in the range of ∼500 to ∼650 Å^2^ in the different compact conformations sampled during the aMD simulation. Interestingly, in the accelerated simulation the 3CD has been seen transiently visiting relatively extended conformations similar to that observed in the crystal structure. The boosting energy in the aMD helped to overcome the interdomain interactions that exist in the compact conformations. From the results of MD simulations it can be concluded that 3CD may exist in solution as a mixture of many compact and extended conformations.

### 3.3. Mutated 3CD has Altered Interface Compared to the WT Protein

Crystal structures of 3C and 3D proteins revealed numerous packing interactions. Mutagenesis was used to prohibit these interactions during crystallization of 3CD. These mutations are E55A, D58A, E63A, C147A, L629D and R638D; see Figure S4. Because some of these substitutions were near the interface of the two domains, this structure provided an opportunity to evaluate the impact of these mutations on the outcome of the simulations. We performed a 50 ns MD simulation of the mutant 3CD and analyzed it in the same manner described for the WT protein; see Experimental section §2.1. Similar to WT simulations, RMSD and radius of gyration analyses indicated large domain movements (Figure S5). Analysis of the conformations sampled during simulations of the mutant protein suggested interdomain interactions between 3C and 3D domains. The interface between the domains in the mutant was found to be different from that observed in the WT protein (Figure S3C).

**Figure 8:**
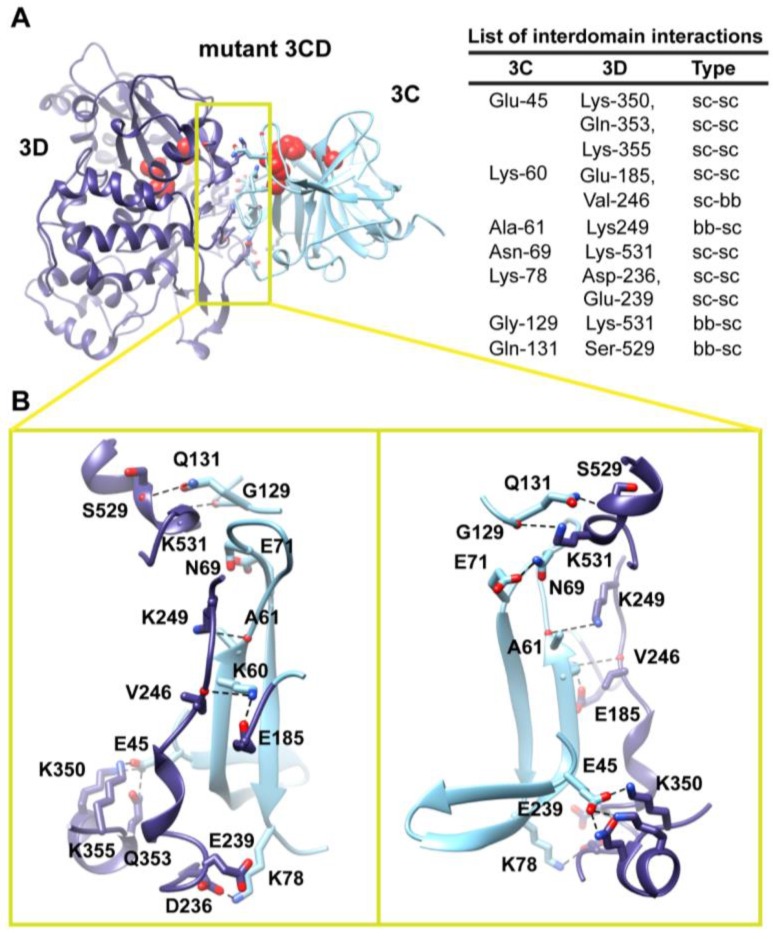
MD simulation reveals altered interface in the mutant 3CD. (**A**) The simulated mutant 3CD is shown with interface residues displayed as sticks; the 3C and 3D domains are colored cyan and blue; respectively. The active-site residues of the protease (His-40, Glu-71, Cys-147) and polymerase (Asp-416, Asp-511, Asp-512) are shown as red spheres to help identifying the relative orientation of the two domains. Interdomain interactions are listed at the right of the panel; two types of interactions are indicated (sidechain-sidechain, sc-sc; sidechain-backbone, sc-bb/bb-sc). (**B**) Two close-up views of the interface marked by the yellow box in (**A**). Interacting residues are shown as sticks and their C-atoms are colored according to the corresponding domains. Interface residues are engaged in many H-bonding and salt bridges, depicted as black dashed-lines. The contact surface between the two domains in the mutant 3CD is different from that observed in the WT protein shown in Figure 5 and Figure 6.

The interdomain interactions found in the mutant 3CD are displayed in Figure 8. The surface area buried between the two domains is ∼700 Å^2^, larger than that observed in the WT protein. Comparison of the interdomain interface of the mutant with that shown in Figure 5 and Figure 6 for WT protein highlighted interesting similarities and differences. Whereas some residues of the 3D-domain at the interface in WT were also present in the mutant (Glu-185, Asp-236, Glu-239, Val-246, Lys-249 and Lys-531), interface residues from the 3C-domain in the mutant were totally different from that in the WT protein. Specifically, the substitution at position-63 in the mutant disrupted the interaction between Glu-63 and Lys-531 observed in the WT protein. The sidechain of Lys-531 in the mutant is engaged in hydrogen bonding interaction with backbone atoms of Gly-129; the aliphatic moiety participates in hydrophobic interactions with that of Asn-69. The sidechain of Asn-69 forms a bridge between the two domains via hydrogen bond formation with the carboxylate of Glu-71. In the vicinity of these interactions, the sidechain of Gln-131 forms a hydrogen bond with the carbonyl oxygen of Ser-529. Additionally, the interactions mediated by the linker region (residues 176–183) in WT are lost in the mutant. The only linker residue that is part of the contact surface in the mutant is Glu-185 of the 3D-domain. It forms a salt bridge with Lys-60, which is hydrogen bonded to the backbone oxygen of Val-246. The environment of Lys-249 in WT is lost in the mutant where it is hydrogen bonded to the backbone oxygen of Ala-61. Lastly, new interactions in the mutant mediated by Glu-45 and Lys-78 have no equivalent in WT. The residue Glu-45 forms two salt bridges with Lys-350 and Lys-355 and a hydrogen bond with the sidechain of Gln-353. Lys-78 forms a salt bridge with Asp-236 and is located within range to participate in electrostatic interactions with Glu-239. We conclude that a single substitution of Glu-63 with alanine at the interface was sufficient to remodel the interface to a completely new conformation.

### 3.4. Experimental SAXS Data Support more than one Conformation for 3CD

The MD simulations revealed a compact conformation with interdomain interactions for 3CD and transiently sampled the extended beads-on-a-string conformation reported in the crystal structure. To see if the two conformations exist and to gain more insight into the solution state, we performed small-angle X-ray scattering (SAXS) experiments; see the Experimental section §2.2. SAXS technique is ideal to explore the large domain movement suggested for 3CD by MD simulations [55].

**Figure 9:**
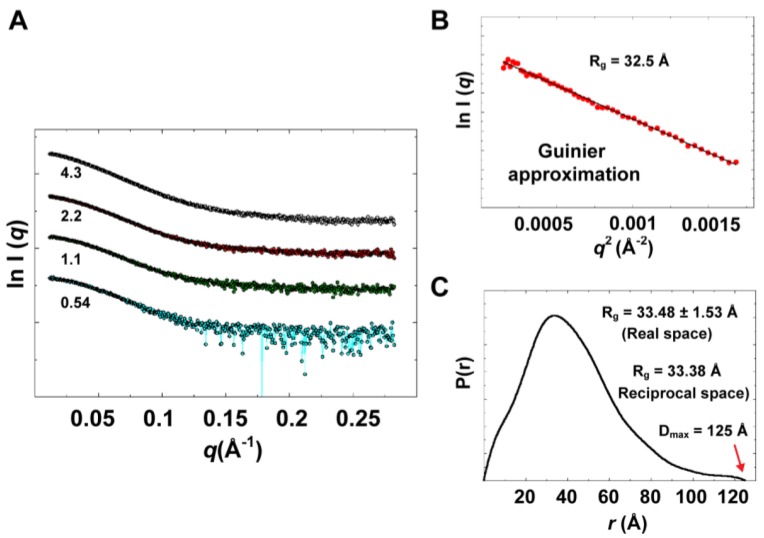
Experimental solution scattering data. (**A**) Shown are plots of the scattering intensity from SAXS data collected at different concentrations of 3CD protein: 0.54 (cyan), 1.1 (green), 2.2 (red) and 4.3 (grey) mg/mL. (**B**) Guinier plot of data at very small angles is shown with a linear regression satisfying the approximation q < 1.3/*R_g_*. (**C**) The pair-distance distribution function P(r) calculated by GNOM is shown. The estimated maximum particle dimension (D_max_) and *R_g_* from P(r) are indicated on the plot. The *R_g_* determined from Gunier approximation is in good agreement with that calculated by GNOM.

Experimental data summary is given in Table 2. SAXS data collected at four different concentrations in the range of 0.54 to 4.3 mg/mL are shown in Figure 9A. Parameters that characterize the size of the protein, radius of gyration (*R*_g_) and maximum particle dimension (D_max_), were determined from data collected at low concentrations (0.54 and 1.1 mg/mL), for which no concentration dependence was observed. Data at very small angles showed linear correlations (Figure 9B) that satisfied Guinier approximation (*qR*_g_ <1.3), from which an *R*_g_ value of 32.5 Å was obtained. The *R*_g_ parameter was also determined from the interatomic pair-distance distribution function P(*r*), computed by GNOM, which takes into account the entire scattering curve, not only the very small-angle portion (Figure 9C). The real-space *R*_g_ obtained from P(*r*) was determined to be 33.48 ± 1.53 Å, which is in good agreement with that obtained from Guinier approximation. It should be noted that the *R*_g_ from SAXS accounts for both protein atoms and solvent molecules in the hydration shell [56]. It is therefore larger than the *R*_g_ calculated from structure coordinates utilizing backbone atoms of the protein during MD simulations**.** The *R*_g_ from SAXS agrees well with the estimated hydrodynamic radius (*R*_h_) from DLS with an *R*_g_/*R*_h_ ratio of 0.86, which suggests a globular shape of 3CD. For comparison, an *R*_g_/*R*_h_ value of ~0.8 is characteristic for globular proteins, the ratio increases as the molecules deviate from globular to elongated shapes, reaching ~1.4 for denatured proteins [57]. The D_max_ parameter was derived from P(*r*) with a value of 125 Å. The estimated size parameters are reasonable for a molecule with the size of 3CD, indicating a monomeric state of the protein in solution. In addition, the molecular masses derived from three different methods (76.9 ± 3.8, 71.5 ± 2.0 and 72.8 ± 2.9 kDa) were consistent with the calculated molecular mass of a monomer (71.92 kDa); see Table 2.

**Table 2:** Small-angle X-ray scattering (SAXS) data analysis for 3CD protein.

Data Collection	
Instrument	G1-line station at CHESS, dual Pilatus 100K-S detector
Beam diameter (μm)	250 × 250
Wavelength (Å)	1.244
*q*-range (Å^−1^)	0.006–0.800
Exposure time (s)	15 × 4
Concentration range (mg mL^−1^)	0.54–4.3
Temperature (K)	293
**Structural parameters**	
*R*_g_ [real-space *R*_g_ from *P*(*r*)] (Å)	33.48 ± 1.53
*R*_g_ (from Guinier) (Å)	32.5
*D*_max_ (Å)	125
**Molecular-mass determination**	
Molecular mass *M_r_* (Da)	
From Porod volume (*V*_p_/1.6)	76,875 ± 3750
From Lysozyme standard	71,492 ± 1953
From SAXS MoW	72,750 ± 2900
Calculated from sequence	71,920
**Software employed**	
Primary data reduction	RAW
Data processing	GNOM
*Ab initio* analysis	DAMMIN, GASBOR
Validation and averaging	DAMAVER
Conformational flexibility	EOM
Computation of model scattering	CRYSOL, FoXS
Fitting structure to SAXS model	SUPCOMB

**Figure 10:**
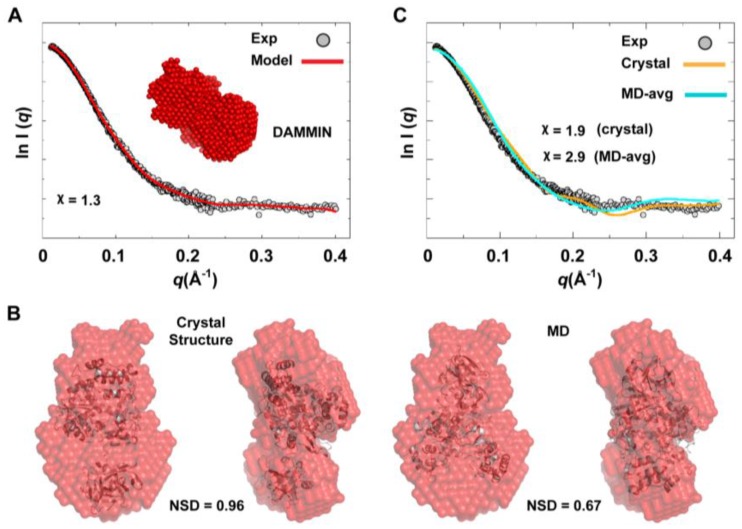
*Ab initio* SAXS model and fitting of single-conformation to experimental scattering data. (**A**) Shown are the average SAXS model constructed using DAMMIN (red spheres) and the fitting of the reference model (red line) to experimental SAXS data (grey). (**B**) The crystal and average MD structures of 3CD, represented as cartoons, are fitted to the DAMMIN model that is shown in A (red transparent surface); two different views are displayed for each fitting. The average MD structure fits the SAXS model slightly better (NSD = 0.67) than the crystal structure (NSD = 0.96). In both cases, however, the exact orientation of the structures cannot be determined without ambiguity. (**C**) The calculated scattering profiles of the crystal and average MD structures are compared to experimental data. The agreement between theoretical profiles and experimental data is relatively poor as indicated by the χ-values, suggesting that no single conformation can satisfactorily fit the experimental data.

We used the combined scattering curve to construct low-resolution SAXS models using DAMMIN and GASBOR programs; see the Experimental section §2.2. Ten independent models were generated from each program and averaged. Some variations among the models were observed as indicated by NSD values that represent their similarity (1.211 ± 0.041 for DAMMIN models and 1.438 ± 0.049 for GASBOR models), which may reflect the flexibility of the protein. The average DAMMIN model is shown in Figure 10A. The model can reasonably fit the two conformations of 3CD: the one observed in the crystal structure and that revealed by MD simulations (Figure 10B). Of note, the structure from simulations showed slightly better fitting (NSD = 0.67) than the crystal structure (NSD = 0.96). Nevertheless, the precise orientation of the protein inside the SAXS envelope could not be determined without ambiguity. Moreover, fitting the calculated scattering profiles of the crystal structure and that of the average MD structure to the experimental curve appeared to be less than optimal with χ values of 1.9 and 2.9 for the crystal structure and the average MD structure, respectively (Figure 10C). This was interpreted to mean that no single conformation can satisfactorily fit the scattering data.

**Figure 11:**
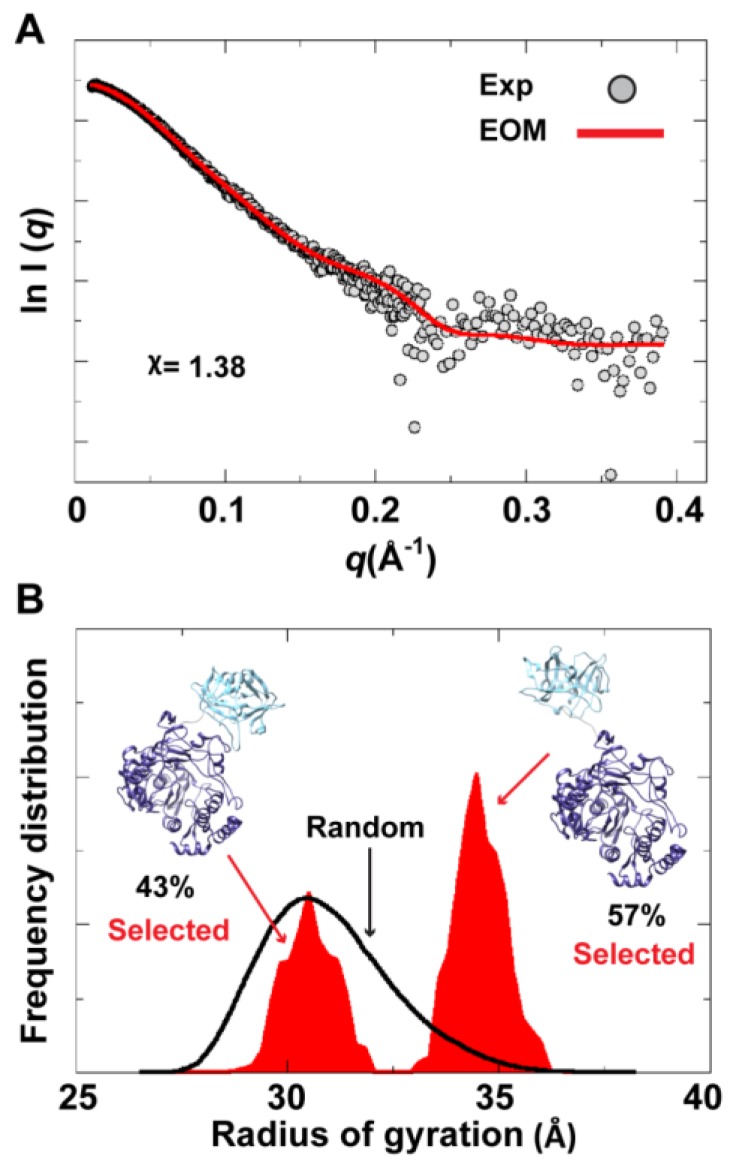
Ensemble Optimization Method (EOM) analysis suggested the presence of two conformations for 3CD in solution. (**A**) The scattering profile calculated from the selected EOM sub-ensemble (red) is in good agreement with the experimental data (grey) as indicated by the low χ-value. (**B**) Shown is the EOM radius of gyration distribution (*R_g_*) of the initial random pool (black line) and the selected sub-ensemble (red shades) for 3CD; the 3C-domain adopts different conformations relative to the 3D-domain in the sub-ensemble. The selected sub-ensemble corresponds to two conformers: an extended conformer that has no interdomain interactions and a compact conformer with interdomain interactions; the extended one is slightly preferred, contributing 57% to the total scattering. The two conformers are displayed in the inset.

To assess the possibility of the presence of multiple conformations for 3CD in solution, we employed the Ensemble Optimization Method (EOM). This method assumes that a mixture of different conformers co-exist in solution and finds the best sub-ensemble out of a randomly generated ensemble consisting of a large number of conformers that best fits the experimental data. From an ensemble of 3CD conformers in which the 3C-domain and the linker residues were allowed to adopt different conformations, EOM selected a sub-ensemble that fits the scattering data better than any single conformation with a χ-value of 1.38 (Figure 11A). Comparison of the *R*_g_ histogram of the initial pool covering the range of 26–38 Å (corresponding to compact-to-extended conformations) with that of the selected sub-ensemble indicated the presence of both extended and compact conformations in solution (Figure 11B). The selected sub-ensemble has a bimodal distribution that could result from switching between the two conformations. The first peak centered at an *R*_g_ of ∼31 Å corresponds to the compact conformation, and the second peak centered at an *R*_g_ of ∼33 Å corresponds to the extended conformation. The conformer corresponding to the first peak accounts for 43% of the total scattering and that corresponding to the second peak accounts for 57%. Thus, both compact and extended conformations are well represented in solution. Of note, the entire initial pool is not represented in the selected sub-ensemble, suggesting a limited conformational space of the protein with the molecule fluctuating around its compact and extended conformations.

In conclusion, the SAXS experiments are consistent with that the conformation of 3CD protein observed in the crystal structure and that revealed by MD simulations co-exist in solution in almost equal proportions. Furthermore, fluctuations around each conformation, similar to what was found in simulations (Figure 4A), could be inferred from the SAXS data.

## 4. Discussion

Biochemical studies of the PV 3CD protein demonstrate quite convincingly that the protein is not merely the sum of its parts (Figure 1). Although 3CD exhibits the 3C-encoded protease activity, the catalytic efficiency and specificity are demonstrably different [13,14]. Even more striking, however, is the fact that 3CD fails to exhibit any of the 3D^pol^-encoded polymerase activity [15]. Observations such as these suggested that the structure of 3CD would be more than two "beads on a string." However, such an organization was observed when the crystal structure of 3CD was reported [23]. The current study was designed to determine if there was more to learn about 3CD than the crystal structure was telling us.

Starting from the crystal structure of 3CD, with modifications to reconstruct the wild-type sequence, molecular dynamics simulations revealed a collapse of the 3C and 3D domains to yield a conformation that could no longer be considered “beads on a string” (Figure 2 and Figure 3). Interestingly, the simulation suggested that the 3C domain can move relative to the 3D domain to create multiple, unique interfaces (Figure 4), although one interface predominated the first trajectory (Figure 5). Performing the same experiment using 3CD coordinates from the second monomer in the unit cell produced the same results as above with respect to the collapse of the 3C and 3D domains (Figure 6). However, the predominating interface observed in this second trial was only partially overlapping with that observed in the first trial. Accelerated molecular dynamics simulations revealed an even wider range of possibilities for the conformations of 3C relative to 3D achievable for 3CD, including the extended conformation observed crystallographically (Figure 7). That the conformational ensemble of 3CD observed computationally exists in solution was shown by using small-angle X-ray scattering experiments (Figure 9, Figure 10 and Figure 11). If each conformation of 3CD exhibits some unique function: catalytic activity or ability to interact specifically with a viral or host factor, then such a conformational ensemble may represent a strategy to expand the viral proteome.

**Figure 12:**
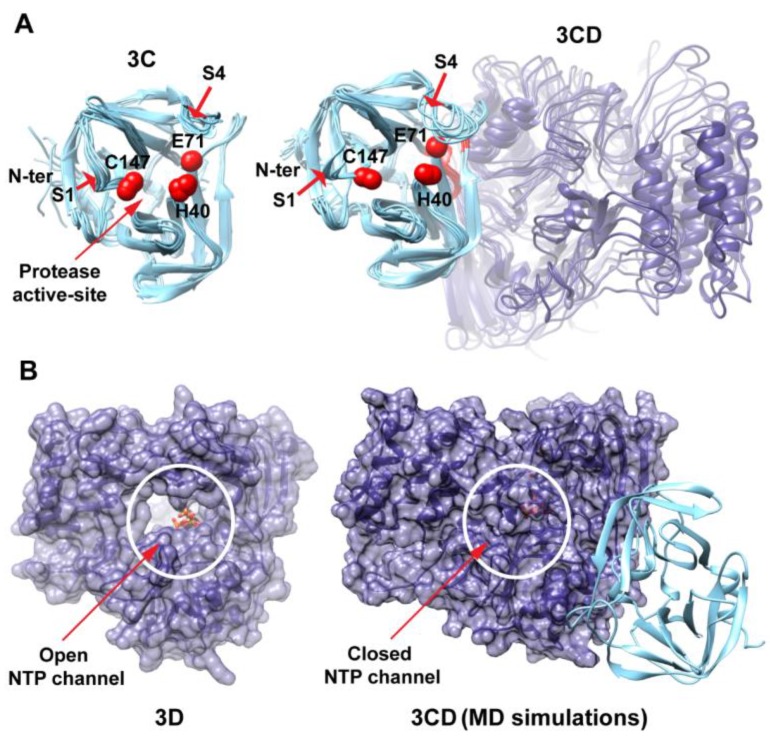
Influence of interdomain interactions on 3CD functions. (**A**) An ensemble of 3C conformations (left) obtained from MD simulations of 3C alone [51] and an ensemble of 3CD conformations (right) are shown. The catalytic triads of the protease active sites are indicated by red spheres; interface residues from 3C-domain in the precursor protein are colored red. MD simulations revealed that the N-terminal residues (aa 1–13), implicated in RNA binding, are much less dynamic in the context of 3CD than 3C alone. Whereas residues implicated in substrate binding or catalysis, including residues of pocket S1 (Thr-142, Gly-145 and Gln-146) and pocket S4 (Leu-125, Leu-127 and Phe-170), are more dynamic in the context of 3CD than 3C alone. These differences in dynamics may contribute to the unique specificity/activity of 3C relative to its precursor protein. Note that the 3D domain in 3CD can participate directly in interactions with polyprotein substrates, which may also contribute to the observed difference between the precursor and processed 3C protein. (**B**) Shown are the crystal structure of PV RdRp with the incoming NTP bound at the active site displayed as sticks (3D, PDB 3OL7 [26]), left, and the average 3CD structure from MD simulations, right. The two structures are oriented using the same view of the polymerase. The 3C and 3D domains are colored cyan and blue, respectively, and the polymerase domain is rendered as transparent surface. The NTP channel is circled and indicated by red arrow. In contrast to 3D structure in which the NTP channel is open, in 3CD the channel is closed and the nucleotide has no access to the active site of the polymerase. Also, interface residues in 3CD maintain interactions with the residues lining the NTP channel, interfering with its opening/closing motion. Additionally, the interdomain interactions in 3CD interfere with functionally important motions of the conserved structural motifs. All these differences in dynamics of 3D alone compared to that in the context of 3CD may contribute to the lack of polymerase activity in the precursor protein.

In addition to revealing the conformational ensemble of the 3CD protein, the molecular dynamics simulations provided insight into the unique specificity/activity of 3CD relative to its domains in isolation. Regarding the proteolytic activity, the organization of the proteolytic active site in the context of 3CD when compared to 3C alone showed no significant difference (Figure 12A) [51]. However, residues of 3C that contribute to substrate binding are more dynamic in the precursor than in the processed protein (Figure 12A). The amino terminus was much more dynamic in the processed protein than in the precursor protein (Figure 12A). Indeed, these differences in dynamics appear as differences in structure when processed and precursor forms are evaluated by X-ray crystallography [23,24]. Of note, it has been shown before that binding of the peptide substrate is accompanied by conformational rearrangements within binding site [24]. Furthermore, portions of the 3D-domain should be able to interact directly with polyprotein substrate (Figure 12A). For the differences in RNA-binding between 3CD and 3C, MD simulations provided plausible insights. Residues of 3C that are implicated in stabilizing the conformation of the RNA recognition site [24] are engaged in interface interactions in the context of 3CD (Figure 5 and Figure 6). These interactions may contribute to the differences in RNA binding activity of 3CD relative to 3C. Additionally, the drastic differences in dynamics of the amino terminus described above in the precursor and processed proteins may also contribute to the differences in RNA binding as these residues are known to be implicated in RNA binding activity [16].

In terms of the missing polymerase activity, it was clear going into this study that the amino terminus penetrates the fingers sub-domain of the polymerase and contributes to the organization of conserved structural motif A, which, in turn, contributes to the organization of conserved structural motif C [25]. Addition of a single amino acid to the amino terminus of the 3D protein reduces polymerase activity by 50–200 fold [37]. Interestingly, in the context of 3CD, the NTP channel is closed (Figure 12B). In the isolated 3D protein, this channel oscillates between open and closed but is most often observed in the open configuration (Figure 12B) [29,58]. This difference may also contribute to the complete absence of polymerase activity associated with 3CD.

We suggest that picornaviruses have evolved 3CD to be devoid of polymerase activity. First, the orthologous protein in the Calicivirus family, Pro-Pol, actually exhibits robust polymerase activity and is thought to be the active form of the enzyme in cells [59,60]. In this system, the role of the amino terminus has been replaced by the addition of an arginine residue to the fingers sub-domain [61]. Second, members of the Flavivirus family encode a methyltranferase domain followed by the polymerase domain [62,63]. In addition, the L (large) proteins of negative strand RNA viruses are all multi-functional, multi-domain proteins exhibiting polymerase activity [64,65].

**Figure 13:**
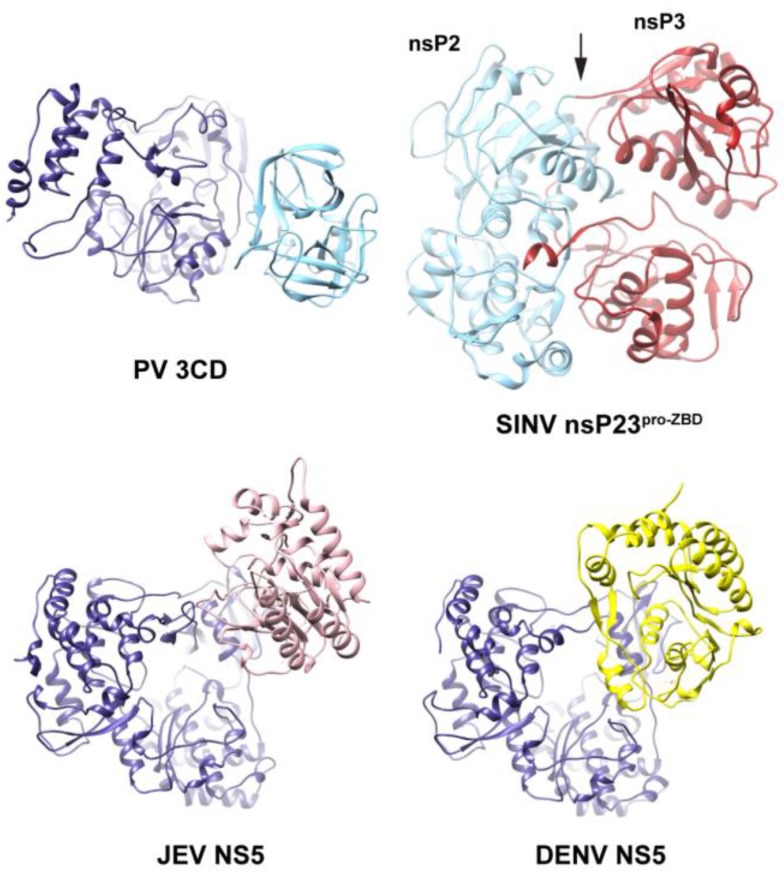
Interdomain interactions in PV 3CD and related proteins. Shown are the simulated structure of PV 3CD, structure of the polyprotein nsP23^pro-ZBD^ from Sindbis virus (SINV, PDB 4GUA), structures of NS5 proteins from Japanese Encephalitis Virus (JEV NS5, PDB 4K6M) and Dengue Virus (DENV NS5, PDB 4V0R). The polymerase domains are colored blue and the N-terminal domains (protease in PV and MTase in NS5) are shown in cyan, pink and yellow. The nsP2 and nsP3 domains are colored cyan and red, respectively. In the four proteins, interfaces between the N- and C-terminal domains exist. The interface in 3CD is a reminiscent of the interfaces observed in the above related proteins. In the structure of nsP23^pro-ZBD^, extensive interdomain interactions between the nsP2 protease and the nsP2/3 domain are maintained. To process this polyprotein, a conformational change is required to expose the cleavage site, suggesting that multiple conformations may exist. In the JEV NS5 structure, the interactions between the N-terminal MTase and the C-terminal polymerase domains are mostly hydrophobic; whereas in DENV NS5 the interdomain interactions are mostly polar. This difference in the nature of the interface suggested that JEV NS5 may possess multiple conformations similar to what has been shown by SAXS for DENV NS5. An interesting difference between 3CD and NS5 is that the linker residues in 3CD (7 amino acids long) are sandwiched between the two domains whereas the linkers in NS5 are not. Note that the linker of JEV NS5 is longer than 3CD (10 amino acids long) and that of DENV NS5 is shorter than 3CD (4 amino acids long). Also, the linker residues in NS5 proteins are less conserved than the linker residues in 3CD.

We speculate that the observation made here for PV 3CD extends to all viral polyprotein precursor proteins. Structures of other precursor or multi-domain viral proteins have been solved (Figure 13) [62,63,66]. These are clearly compact structures with interdomain interactions. While solution information is not available for the alphavirus nsP23 precursor, SAXS experiments have been performed for the flavivirus NS5 protein [67]. These experiments suggest a conformational ensemble for NS5 as well. There is a clear need to place greater emphasis on the dynamics and conformational sampling of viral proteins, both fully processed and precursor forms, in the future.

Our laboratory has been quite intrigued by the concept that RNA viruses need to create mechanisms to expand their functional proteome [68]. This notion emerged when considering hepatitis C virus (HCV). HCV encodes fewer than one dozen proteins but is capable of precluding clearance by the human body as long as the liver can tolerate infection, usually many decades. We have provided very compelling evidence that in the case of HCV the use of an intrinsically disordered protein whose structure and dynamics can be regulated by phosphorylation is the strategy this virus uses to expand its functional proteome. Acute RNA viruses likely require more function than they encode using traditional approaches but not as many as persistent viruses. Use of the conformational ensemble is quite elegant. Even more elegant is the ability to make this ensemble sensitive to single amino acid substitutions. RNA viruses exist as a population of genetic variants, with the possibility for each amino acid to be sampled at each position of each protein. Our studies show that a single amino acid substitution in the interdomain interface of a precursor protein can completely change the architecture of the interface and the ensemble of conformations present and/or populated (Figure 8). Whether or not the conformational ensemble of multi-domain viral proteins as suggested here is a trait unique to this class of viral proteins, or cellular proteins as well, merits further investigation.

## 5. Conclusions

The use of a polyprotein for gene expression has always been considered a strategy to encode more functions into the limited coding capacity of a virus. Here we suggest that conformational sampling of interdomain interactions in polyprotein processing intermediates may further increase the viral proteome. The impact of mutation at the interdomain interfaces can be transformative to the conformational ensemble and hence the proteome, providing a possible explanation for the requirement of genetic diversity of an RNA virus for optimal fitness.

## Supplementary Files

Supplementary File 1
