# Correction: Moustafa, I.M.; *et al.* Conformational Ensemble of the Poliovirus 3CD Precursor Observed by MD Simulations and Confirmed by SAXS: A Strategy to Expand the Viral Proteome? Viruses 2015, 7, 5962–5986 

**DOI:** 10.3390/v7122954

**Published:** 2015-12-11

**Authors:** Viruses Editorial Office

**Affiliations:** MDPI AG, Klybeckstrasse 64, CH-4057 Basel, Switzerland; viruses@mdpi.com

The *Viruses* Editorial Office wishes to add the section “Supplementary Materials” to [1]. 

**Supplementary Materials:** The following are available online at http://www.mdpi.com/1999-4915/7/11/2919, Figure S1: DLS analysis of PV 3CD protein, Figure S2: PCA analysis reveal major motions of 3CD, Figure S3: MD simulations reveal multiple interfaces in WT and mutant 3CD, Figure S4: Mutations of 3CD in the crystal structure, Figure S5: Analysis of the 50 ns MD simulations of mutant PV 3CD, and Video S1: Conformational Ensemble of the Poliovirus 3CD Precursor Observed by MD Simulations and Confirmed by SAXS: A Strategy to Expand the Viral Proteome?; DOI: 10.5281/zenodo.34538.

## References

[1] Moustafa I.M., Gohara D.W., Uchida A., Yennawar N., Cameron C.E. (2015). Conformational Ensemble of the Poliovirus 3CD Precursor Observed by MD Simulations and Confirmed by SAXS: A Strategy to Expand the Viral Proteome?. Viruses.

