# Supplementary material for: Conformational Ensemble of the Poliovirus 3CD Precursor Observed by MD Simulations and Confirmed by SAXS: A Strategy to Expand the Viral Proteome?
Source: Viruses. 2015 Nov 23;7(11):5962–86. doi: 10.3390/v7112919 (PMC4664992; doi:10.3390/v7112919)
Supplement: Supplementary File 1 [file viruses-07-02919-s001.zip › viruses-88963-supplementary revised.pdf]

Supplemental Information

Conformational ensemble of the poliovirus 3CD precursor observed by MD simulations and confirmed by SAXS: A strategy to expand the viral proteome?

Ibrahim M. Moustafa [1*], David W. Gohara [2], Akira Uchida [1], Neela Yennawar [3] and Craig E. Cameron [1,*]

[1] Department of Biochemistry and Molecular Biology, The Pennsylvania State University, University Park, PA 16802, USA

[2] Department of Biochemistry and Molecular Biology, St Louis University School of Medicine, 1100 South Grand Ave, St Louis, MO 63104, USA

[3] Huck Institutes of life sciences, The Pennsylvania State University, University Park, PA 16802, USA

***** Author to whom correspondence should be addressed; E-Mail: cec9@psu.edu & ria2@psu.edu ; Tel.: +1-814-863-8705

Figure S1

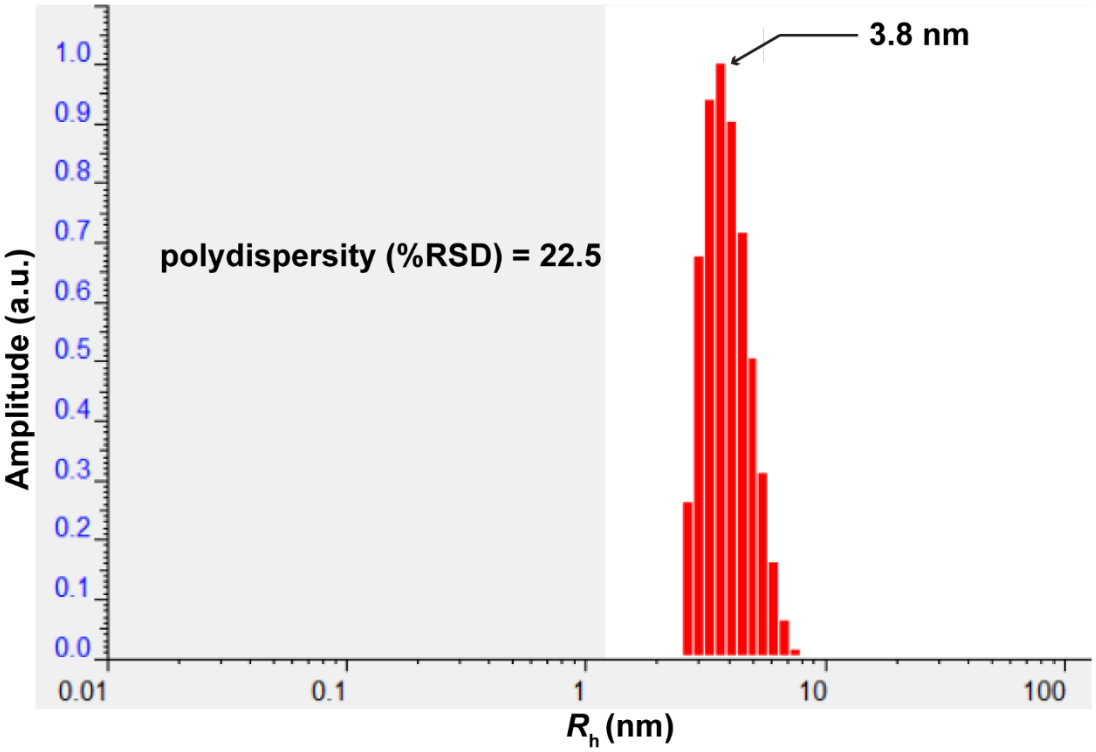

Figure S1. DLS analysis of PV 3CD protein. DLS measurement for the protein sample (2.0 mg/ml) is shown. The hydrodynamic radius (R_h) was estimated to be 3.8 nm and the polydispersity of the sample was estimated to be 22.5%.

Figure S2

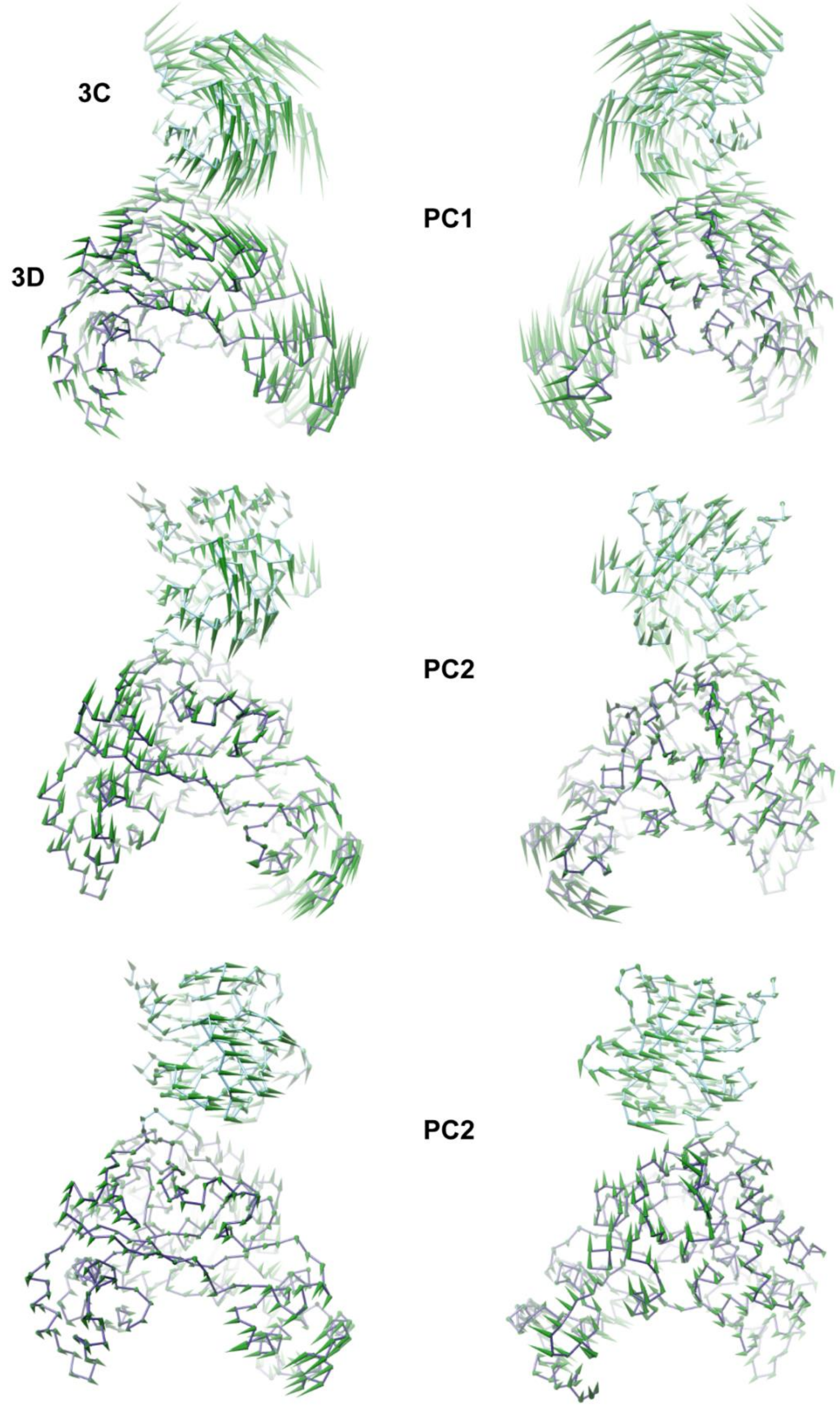

Figure S2. PCA analysis reveal major motions of 3CD. The motion contents of the largest three principal components (or modes) PC1, PC2, and PC3 of PCA are visualized in two views

for each mode. The average 3CD is depicted as wires with 3C and 3D domains colored cyan and blue, respectively. The directions of displacements of Cα atoms in the different modes are indicated by green "porcupine needles"; the size of the needle is proportional to the displacement magnitude. From the directions of the displacement vectors, the rigid-body motions of the 3C-domain and the thumb subdomain of the polymerase can be identified.

Figure S3

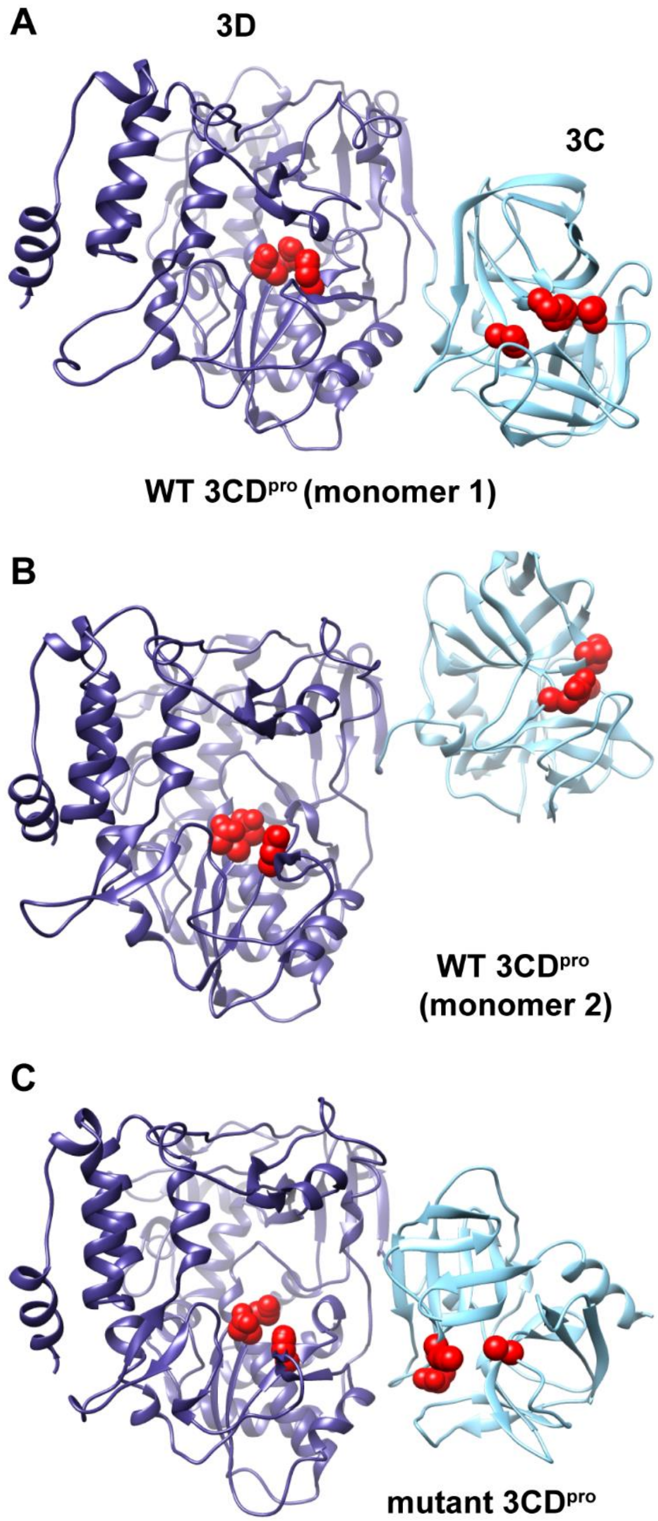
A
3D
3C
WT 3CD^{pro} (monomer 1)
B
WT 3CD^{pro}
(monomer 2)
C
mutant 3CD^{pro}

Figure S3. MD simulations reveal multiple interfaces in WT and mutant 3CD. Shown are the calculated average structures from MD simulations of WT (monomer 1) (A), WT (monomer 2) (B), and mutant 3CD (C); 3C and 3D domains are colored cyan and blue, respectively. The active-site residues of the protease (His-40, Glu-71, Cys-147) and polymerase (Asp-416, Asp-511, Asp-512, 3CD numbering) are shown as red spheres to indicate the relative orientation of the two domains. The views in A-C display the 3D domains in the same orientation; the orientations of the 3C-domain relative to the 3D-domain in the WT and mutant proteins are different.

Figure S4

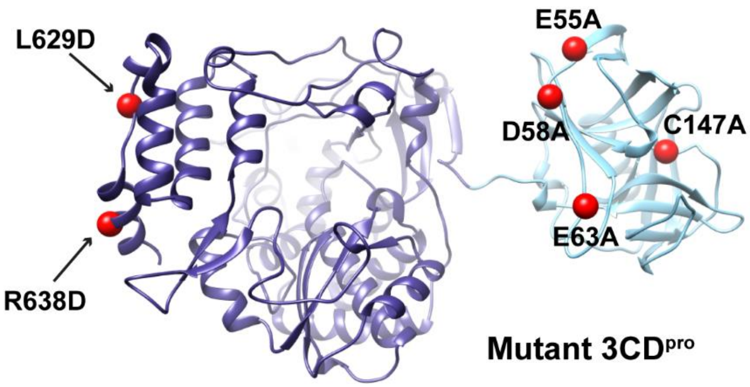

Figure S4. Mutations of 3CD in the crystal structure. Crystal structure of PV 3CD with the mutations introduced to facilitate crystallization process mapped onto the structure and represented as red spheres. The 3C and 3D domains are colored cyan and blue, respectively.

Figure S5

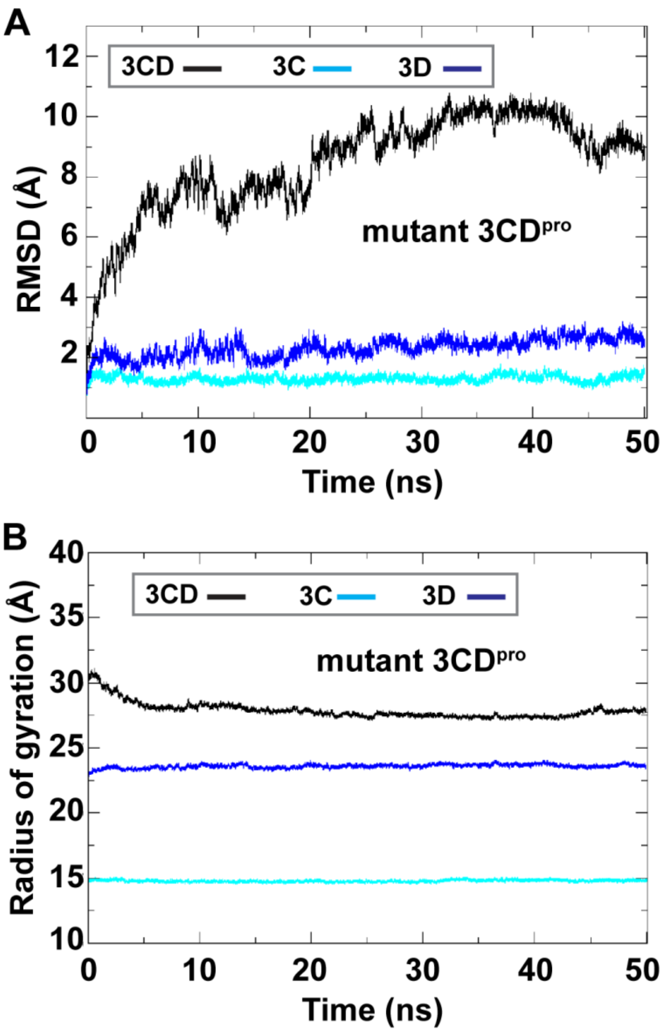

Figure S5. Analysis of the 50 ns MD simulations of mutant PV 3CD. Shown are the RMSD (A) and radius of gyration R_g (B) analyses of the MD trajectory of the mutant 3CD. Similar to the results obtained from simulations of WT 3CD described in the main text and shown in Figures 2 and 3, the analyzed MD trajectory of the mutant 3CD reveal large domain movements during the simulations.
